# Optimizing Functional Performance in Sprengel’s Shoulder: A Case Report

**DOI:** 10.7759/cureus.110441

**Published:** 2026-06-08

**Authors:** Siddhi P Patrekar, Sandeep Shinde, Sakshant Desai, Vaibhav V Shinde

**Affiliations:** 1 Department of Musculoskeletal Sciences, Krishna College of Physiotherapy, Krishna Vishwa Vidyapeeth, Deemed To Be University, Karad, IND

**Keywords:** functional performance, functional rehabilitation, physiotherapy rehabilitation, shoulder dysfunction, sprengel’s deformity, sprengel's shoulder, upper-limb function

## Abstract

Sprengel's deformity is a rare congenital musculoskeletal condition characterized by elevation and malposition of the scapula, leading to cosmetic deformity and functional limitations of the shoulder complex. The condition is commonly associated with restricted scapulothoracic mobility, reduced glenohumeral range of motion, muscle weakness, and impaired upper-limb function, which may affect activities of daily living and quality of life. This case report describes the clinical presentation, radiological findings, and physiotherapy management of a patient with Sprengel's shoulder.

A structured six-week physiotherapy rehabilitation program was implemented to improve shoulder mobility, muscle strength, scapular control, and functional performance. The intervention was delivered progressively in three phases: pain management and mobility restoration; stretching, postural correction, and isometric strengthening; and advanced resistance training, scapular stabilization, proprioceptive facilitation, and task-oriented functional activities.

Following the intervention, significant improvements were observed in shoulder function. Manual Muscle Testing (MMT) grades improved from 3/5 to 4/5 in the upper trapezius, middle trapezius, lower trapezius, rhomboids, serratus anterior, and levator scapulae muscles. Active shoulder flexion increased from 120° to 160°, while shoulder abduction improved from 110° to 155°. Functional disability, assessed using the Disabilities of the Arm, Shoulder and Hand (DASH) questionnaire, improved from 32.5/100 to 12.5/100. Enhanced scapular control, postural alignment, overhead reaching ability, and overall upper-extremity function were also noted.

This case highlights the potential benefits of early, individualized, and progressive physiotherapy rehabilitation in improving functional outcomes in individuals with Sprengel's deformity.

## Introduction

The shoulder complex is a highly mobile musculoskeletal system comprising the glenohumeral, acromioclavicular, sternoclavicular, and scapulothoracic articulations. The coordinated interaction of these structures enables movement in multiple planes and provides the upper extremity with a wide range of motion necessary for functional activities [1]. Shoulder disorders are a common source of musculoskeletal pain and functional limitation in the general population. Epidemiological studies have identified the shoulder as one of the most frequently affected anatomical regions, contributing substantially to disability and reduced quality of life [2].

Sprengel’s deformity (SD) or Sprengel’s shoulder is an uncommon shoulder girdle malformation whose cause is unclear. In this deformity, the defining hallmark is the elevation of the scapula. During normal embryological development, the scapula originates in the cervical region opposite the fourth to sixth cervical vertebrae and gradually descends to its normal thoracic position between the second and seventh thoracic vertebrae by the end of fetal development. Sprengel's deformity is believed to result from failure or incomplete descent of the scapula during this embryological process, leading to congenital elevation and malposition of the scapula. The presenting finding is a lump at the back of the neck with limited shoulder and/or arm movement. On its sagittal plane, the afflicted scapula is rotated. The medial line and the axilla are closer to the superior and vertebral borders, respectively [3]. According to traditional research on SD, the scapula is hypoplastic, frequently smaller than the normal side, and the ratio of horizontal to vertical diameter is higher. The trapezius, levator scapulae, and rhomboids-the muscles that move the scapula-are frequently hypoplastic or invaded with fibrofatty tissue [4].

The scapula may be raised by 2 to 10 cm and adducted in SD, with its lower angle turned medially and frequently nearing the midline. The primary clinical changes are incorrect scapular posture and hypoplasia, which can lead to both cosmetic problems and shoulder girdle movement limits [5].

Although cosmetic correction is often considered a primary goal of surgical treatment for Sprengel's deformity, surgical intervention may also improve shoulder function by enhancing scapular position, increasing shoulder range of motion, and reducing biomechanical limitations. The extent of functional improvement varies depending on the severity of the deformity, associated anomalies, and the surgical technique employed. Consequently, several surgical techniques have been developed to improve functional results [6].

Although Sprengel's deformity is a rare congenital condition, most published literature has focused on surgical correction, while evidence regarding conservative physiotherapy management remains limited. Patients with mild deformities often present with functional limitations, altered scapular mechanics, and reduced upper-limb performance that may be managed non-operatively. Therefore, documenting the outcomes of a structured physiotherapy rehabilitation program is important to enhance the understanding of conservative management strategies [7]. The rationale for this case report was to evaluate the effectiveness of a progressive physiotherapy rehabilitation program in improving shoulder mobility, muscle strength, scapular control, and functional performance in a patient with mild Sprengel's deformity.

## Case presentation

A 21-year-old male patient presented with complaints of asymmetry of the shoulder girdle, difficulty performing overhead shoulder activities, and reduced functional performance of the affected upper limb during activities of daily living. The patient’s height was 170 cm, weight was 65 kg, and body mass index (BMI) was 22.5 kg/m². He reported no severe pain but expressed cosmetic concern and limitation in shoulder movement.

According to the patient's history, mild shoulder asymmetry had been noticed since childhood; however, no medical consultation was sought initially because the deformity was minimal and did not significantly affect function during the early years. At approximately 17 years of age, the patient began experiencing increasing difficulty with overhead activities, lifting tasks, prolonged upper-limb use, and certain daily functional activities. Following medical consultation, clinical and radiological evaluation confirmed the diagnosis of Sprengel's deformity (SD). The patient was later referred for physiotherapy rehabilitation due to persistent functional limitations and altered scapular mechanics. The patient had no relevant past surgical history, no history of trauma to the shoulder region, and no associated neurological disorders. Family history was non-contributory, with no known congenital musculoskeletal abnormalities among close relatives. Developmental milestones during childhood were reported to be normal.

Clinical examination revealed elevation of the left scapula with mild restriction of the shoulder range of motion. Baseline goniometric assessment demonstrated active shoulder flexion of 120°, extension of 40°, abduction of 110°, and adduction of 25° on the affected side. Manual Muscle Testing (MMT) revealed Grade 3/5 strength in the upper trapezius, middle trapezius, lower trapezius, rhomboids, serratus anterior, and levator scapulae muscles. The patient also exhibited impaired scapulothoracic rhythm and difficulty performing overhead functional activities. The patient also demonstrated weakness of the periscapular muscles and reduced functional performance of the affected upper limb during activities of daily living. On observation, a slight asymmetry of the shoulders was noted, with the affected scapula positioned higher than the opposite side. The deformity was minimal and not clearly visible when the patient was fully clothed. Based on clinical appearance, the deformity was classified as Grade I according to the Cavendish classification system. This grading was based on clinical observation of a very mild elevation of the left scapula that was not readily visible when the patient was fully dressed and did not result in significant cosmetic deformity. Although a slight asymmetry of the shoulder girdle was present, there was no obvious neck webbing, marked scapular elevation, or severe shoulder imbalance. These findings were consistent with the objective criteria for Cavendish Grade I (very mild deformity), in which the deformity is minimal and often detected only on careful physical examination.

Radiological evaluation using anteroposterior chest and shoulder radiographs confirmed superior displacement and malposition of the left scapula, consistent with Sprengel's deformity. The affected scapula was positioned higher than the contralateral side, with no evidence of associated vertebral anomalies, rib abnormalities, or other significant skeletal deformities. Radiographic findings correlated with the clinical classification of Cavendish Grade I deformity, characterized by mild scapular elevation and minimal cosmetic involvement. No omovertebral bone was identified on imaging. Physiotherapy assessment demonstrated decreased active shoulder flexion and abduction, tightness of the upper trapezius and levator scapulae muscles, reduced scapular mobility, and mild weakness of the shoulder stabilizers. Functional limitations were mainly observed during overhead reaching, lifting activities, and prolonged upper-limb use.

Written informed consent was obtained from the patient for participation in the study, and the patient was assured that all personal information would be kept confidential. The patient underwent a structured physiotherapy rehabilitation program conducted six sessions per week for six weeks, with each session lasting approximately 45-60 minutes. Focusing on pain reduction, mobility restoration, stretching, strengthening, scapular stabilization, postural correction, and functional retraining. Progressive improvement was observed in shoulder range of motion, muscle strength, scapular control, and overall upper-extremity function following the rehabilitation protocol. SD is the most prevalent congenital deformity of the shoulder girdle, yet it remains uncommon. The prevalence of the deformity is reported to be three times higher in men than in women. Although unilateral SD is most common, with a preference for the left side, bilateral cases may occur and are often associated with greater functional limitation and less visible deformity than unilateral cases.

**Figure 1:**
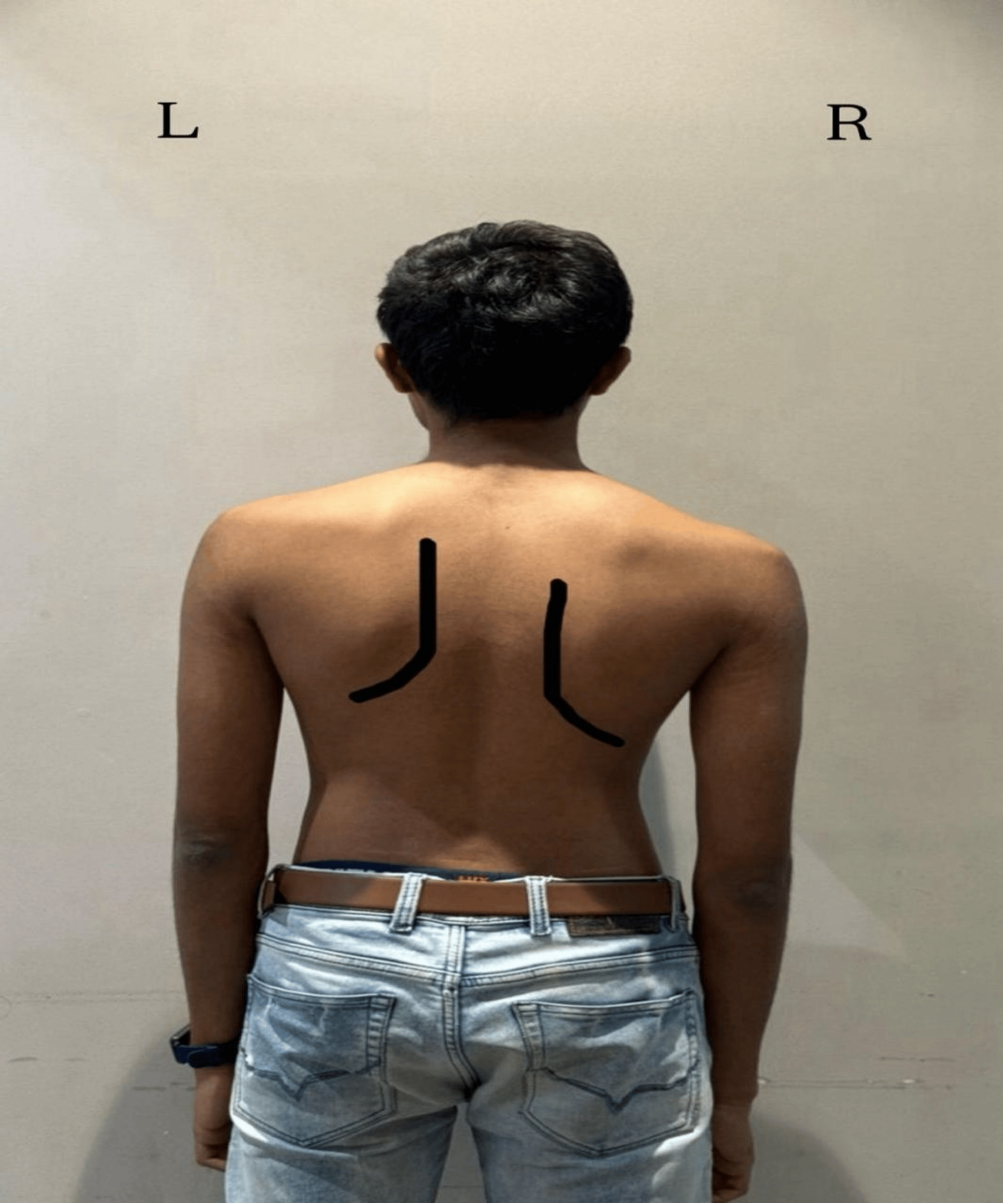
Asymmetry of the shoulder girdles, with the higher position of the left scapula and asymmetry in the position of the inferior scapular poles (dashed lines) L: left; R: right

Functional impairment and cosmetic deformity caused by shoulder girdle asymmetry are the primary complaints in SD. Reduced glenohumeral abduction results from restricted scapulothoracic mobility, which is often relatively well tolerated, as given in Figure 1.

**Figure 2:**
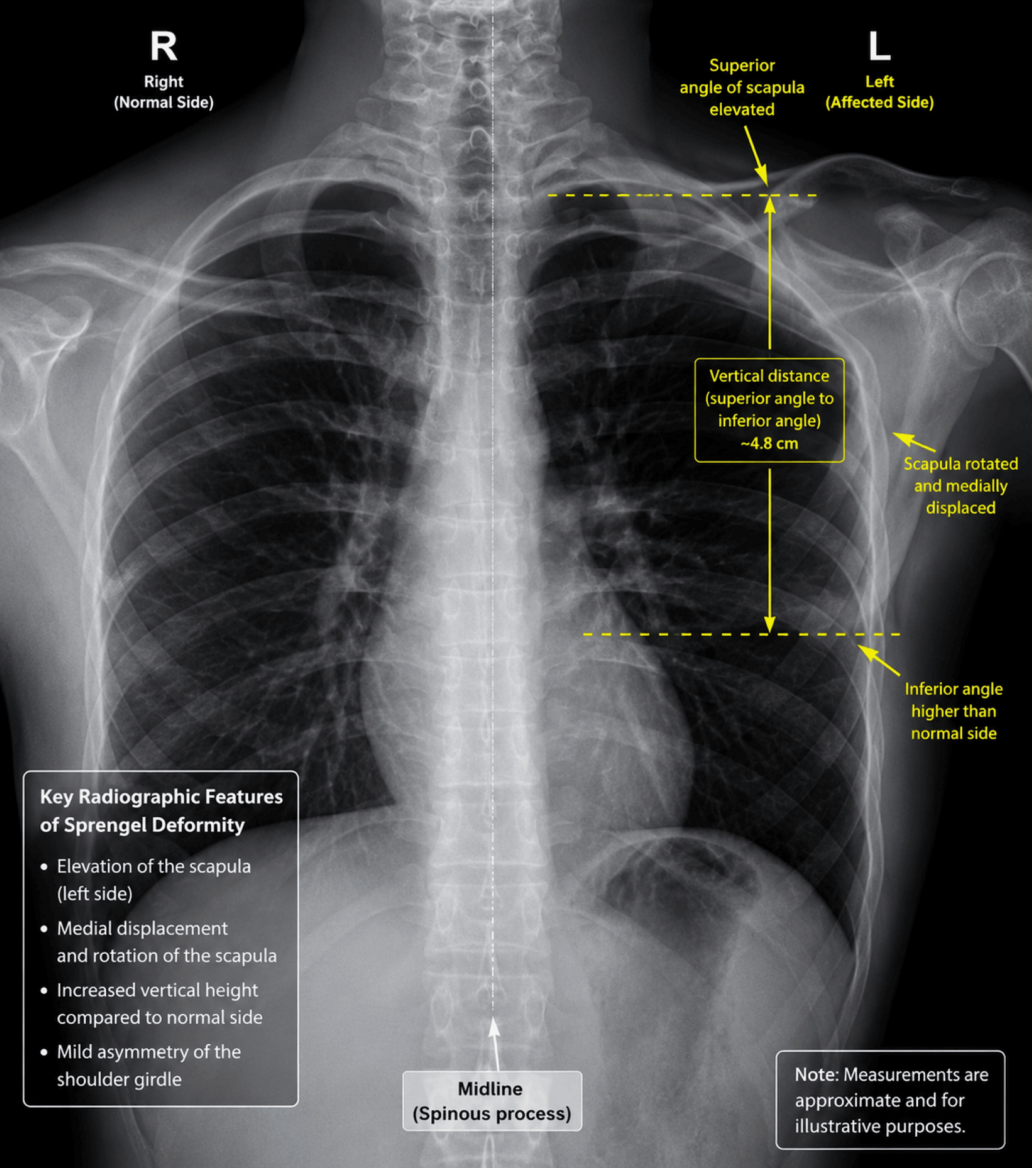
Chest radiograph showing elevated left scapula consistent with Sprengel's deformity and mild asymmetry of the shoulder girdle

The radiograph demonstrates elevation of the left scapula relative to the contralateral side, consistent with Sprengel's deformity. The affected scapula is positioned higher within the thoracic cage and exhibits mild medial displacement and rotational malalignment. Arrows indicate the elevated superior angle and altered scapular position. Dashed reference lines highlight the asymmetry between the left and right scapulae, illustrating the difference in scapular height. No obvious omovertebral bone or other major associated skeletal abnormalities are identified on this radiograph. These findings are consistent with a mild (Cavendish Grade I) Sprengel's deformity and correlate with the clinical findings of shoulder asymmetry, restricted shoulder motion, and altered scapulothoracic mechanics, as given in Figure 2.

Physiotherapy treatment

The physiotherapy rehabilitation program for the patient with SD was planned progressively over a period of six weeks with the aim of improving shoulder mobility, muscle strength, scapular stability, and functional performance of the upper limb. The patient demonstrated improvement in shoulder range of motion, muscle strength, scapular positioning, and overall functional performance following completion of the physiotherapy rehabilitation program, as given in Table 1.

**Table 1:** Physiotherapy treatment PROM: passive range of motion; AAROM: active assisted range of motion; PNF: proprioceptive neuromuscular facilitation; ADL: activity of daily living; Reps: repetition; Sec: second

Week	Exercises	Frequency	Phase/rationale
Week 1	Deep breathing exercises, PROM, shoulder shrugs, retraction/protraction, rotation	10 reps × 1 set	Pain reduction, mobility restoration
Week 2	PROM, pendulum exercises, AAROM, finger ladder, upper trapezius stretch	10 reps × 1 set; stretch 10 sec × 5 reps	Joint mobility, flexibility, initiation of active movement
Week 3	AAROM, finger ladder, levator scapulae stretch, pectoralis stretch	10 reps × 1 set; stretch 10 sec × 5 reps	Flexibility improvement, postural correction, mobility enhancement
Week 4	Isometric exercises, wand exercises, light TheraBand resistance	10 reps × 1 set; isometrics 10 sec × 5 reps	Early strengthening, muscular activation, scapular control
Week 5	TheraBand strengthening, resisted exercises, wall push-ups, scapular stabilization drills	10 reps × 1 set	Strengthening scapular stabilization
Week 6	Advanced TheraBand PNF diagonals, overhead activities, ADL training	10 reps × 1 set	Functional strengthening, coordination, task-specific retraining, and return to daily activities

Result

Pain intensity assessed using the Numeric Pain Rating Scale (NPRS) demonstrated improvement following rehabilitation. NPRS at rest decreased from 2/10 to 0/10, while NPRS during activity decreased from 5/10 to 1/10. These findings indicate a reduction in pain severity and improved tolerance for functional and overhead activities, as given in Table 2.

**Table 2:** Numeric Pain Rating Scale NPRS: Numeric Pain Rating Scale

Outcome measure	Pre-intervention	Post-intervention
NPRS at rest (0–10)	2/10	0/10
NPRS during activity (0–10)	5/10	1/10

Functional disability assessed using the Disabilities of the Arm, Shoulder and Hand (DASH) questionnaire improved following the six-week physiotherapy rehabilitation program. The DASH score decreased from 32.5/100 pre-intervention to 12.5/100 post-intervention, representing an improvement of 20 points. A reduction of approximately 20 points is generally considered clinically meaningful and is consistent with the observed improvements in shoulder range of motion, periscapular muscle strength, scapular control, overhead functional activities, and activities of daily living following rehabilitation, as given in Table 3.

**Table 3:** DASH score DASH: Disabilities of the Arm, Shoulder, and Hand

Outcome measure	Pre-intervention	Post-intervention
DASH score (0–100)	32.5	12.5
Change score	–	20.0 points improvement

Goniometric assessment demonstrated improvements in all measured shoulder movements following the six-week physiotherapy rehabilitation program. Active shoulder flexion improved from 120° to 160°, extension from 40° to 55°, abduction from 110° to 155°, and adduction from 25° to 40°, indicating enhanced shoulder mobility and functional capacity, as given in Table 4.

**Table 4:** Range of motion

Movement	Pre-intervention (°)	Post-intervention (°)	Improvement (°)
Flexion	120	160	+40
Extension	40	55	+15
Abduction	110	155	+45
Adduction	25	40	+15

Post-intervention assessment demonstrated improvement in muscle strength of the periscapular musculature on Manual Muscle Testing (MMT). The trapezius, rhomboids, and serratus anterior muscles improved from Grade 3/5 at baseline to Grade 4/5 following the 6-week rehabilitation program. Improvement was also observed in scapulothoracic control, posture, and performance of overhead functional activities, as given in Table 5.

**Table 5:** Manual Muscle Testing MMT: Manual Muscle Testing

Muscle tested	Pre-intervention MMT grade	Post-intervention MMT grade
Upper trapezius	3/5	4/5
Middle trapezius	3/5	4/5
Lower trapezius	3/5	4/5
Rhomboids	3/5	4/5
Serratus anterior	3/5	4/5
Levator scapulae	3/5	4/5

## Discussion

The present case highlights the importance of a structured physiotherapy rehabilitation program in managing functional limitations associated with Sprengel's deformity (SD). SD is a congenital condition characterized by abnormal elevation and malposition of the scapula resulting from failure of normal scapular descent during embryological development [7,8]. Patients commonly present with cosmetic deformity, restricted shoulder movement, altered scapulothoracic mechanics, and weakness of the periscapular musculature, all of which can negatively affect upper limb function and quality of life [9].

Radiological assessment plays a significant role in confirming the diagnosis and evaluating the severity of SD. Frontal radiographs are useful in identifying scapular elevation and determining the relationship of the superomedial angle of the scapula with the vertebral column [10]. Oblique radiographic views may additionally reveal the presence of an omovertebral bone connecting the scapula to the cervical spine. Other radiographic findings include rotational asymmetry of the scapulae, anterior curvature of the supraspinous scapular region, and altered scapular positioning [11,12]. Scapular medialization can also be measured using the distance between the medial border of the scapula and a vertical line passing through the C7 spinous process. Although less commonly used, ultrasonography may help identify cartilaginous or fibrous bands, while MRI is beneficial in detecting associated spinal cord abnormalities such as diastematomyelia and soft tissue anomalies [12].

The clinical severity of SD is most commonly assessed using the Cavendish classification system [13]. This categorizes deformity according to cosmetic appearance and degree of scapular elevation. Grade I represents a very mild deformity with almost symmetrical shoulders and no visible abnormality while clothed, whereas Grade IV indicates severe deformity with the superior angle of the scapula positioned near the occiput, along with marked functional limitation. In the present case, the patient was classified as Grade I according to the Cavendish classification due to minimal scapular elevation, mild asymmetry, and negligible cosmetic involvement. This mild clinical presentation may explain the favorable response to conservative physiotherapy management.

Pandey et al. reported successful management of moderate-to-severe Sprengel's deformity using the modified Woodward surgical procedure in pediatric patients with Cavendish Grade III deformity, demonstrating improvement in scapular alignment, cosmetic appearance, and shoulder range of motion during long-term follow-up. Their study primarily focused on operative correction of structural deformity and emphasized that early surgical intervention may lead to better functional outcomes [14]. In comparison, the present case report highlights the effectiveness of conservative physiotherapy rehabilitation in a patient with mild Cavendish Grade I deformity. Unlike the surgical approach described by Pandey et al., the current study employed a structured six-week physiotherapy protocol comprising progressive mobility exercises, stretching, strengthening, scapular stabilisation, proprioceptive neuromuscular facilitation, and functional retraining [14]. The present case demonstrated improvement in periscapular muscle strength, scapulothoracic control, posture, and overhead functional performance following rehabilitation. While the surgical study mainly addressed cosmetic and anatomical correction, the current article provides greater emphasis on functional recovery, neuromuscular control, and rehabilitation-based management, suggesting that individualized physiotherapy interventions may play an important role in optimizing upper-limb function and quality of life in patients with mild SD.

The rehabilitation protocol followed a progressive approach aimed at restoring mobility, improving muscular strength, and enhancing functional shoulder mechanics. During the early phase of rehabilitation, interventions such as passive range of motion (ROM) exercises, pendulum exercises, breathing exercises, and active-assisted movements were implemented to reduce stiffness, improve circulation, and initiate neuromuscular activation. Early mobilization may have contributed to the maintenance of joint flexibility and the prevention of secondary soft tissue tightness.

The intermediate phase focused on stretching shortened musculature and improving muscle activation through isometric strengthening exercises. Tightness in the upper trapezius, levator scapulae, and pectoral muscles is commonly observed in SD due to abnormal scapular positioning. Therefore, targeted stretching combined with controlled strengthening exercises likely improved scapular mobility and muscular coordination. The gradual addition of resistance exercises using TheraBand (Performance Health Headquarters, Akron, Ohio, USA), further enhanced the dynamic stabilization of the shoulder girdle.

In the final phase of rehabilitation, emphasis was placed on functional strengthening and scapular stabilization. Exercises such as prone I, Y, T, and W raise, wall push-ups, and proprioceptive neuromuscular facilitation (PNF) diagonal patterns were included to improve scapulohumeral rhythm, muscular endurance, and upper limb coordination. These interventions are essential for restoring normal scapular mechanics and improving efficiency during overhead activities and activities of daily living. Functional retraining may also have contributed to improved postural alignment and reduction of compensatory movement patterns.

The present case demonstrated measurable improvements following six weeks of physiotherapy rehabilitation. Manual Muscle Testing (MMT) [15], grades increased from 3/5 to 4/5 in the upper trapezius, middle trapezius, lower trapezius, rhomboids, serratus anterior, and levator scapulae muscles. Shoulder range of motion also improved, with flexion increasing from 120° to 160°, extension from 40° to 55°, abduction from 110° to 155°, and adduction from 25° to 40°. Functional disability, assessed using the Disabilities of the Arm, Shoulder and Hand (DASH) questionnaire [16], improved from 32.5/100 to 12.5/100, while pain intensity measured using the Numeric Pain Rating Scale (NPRS) during activity decreased from 5/10 to 1/10. These objective findings suggest that a structured physiotherapy rehabilitation program may contribute to improved muscle strength, shoulder mobility, pain reduction, and upper-extremity function in individuals with mild Sprengel's deformity.

The present study mainly focused on improving functional performance associated with Sprengel's deformity. The functional improvements observed after the physiotherapy rehabilitation program included enhancement of shoulder range of motion, scapulothoracic mobility, scapular stability, muscular strength, posture, and upper-limb coordination. Specifically, the patient demonstrated improved overhead reaching ability, lifting performance, activities of daily living (ADL), and scapulohumeral rhythm following intervention. Improvement in periscapular muscle strength was observed in the trapezius, rhomboids, serratus anterior, and levator scapulae muscles, contributing to better scapular control and reduced compensatory movements. Additionally, the rehabilitation program improved neuromuscular activation, postural alignment, functional shoulder mechanics, and overall upper-extremity performance, thereby enhancing independence and quality of life in the patient with SD.

Overall, the findings of this case suggest that a progressive physiotherapy program emphasizing mobility restoration, scapular stabilization, strengthening, and functional retraining may effectively improve functional outcomes in individuals with SD. However, conservative management primarily addresses functional limitations, while structural deformity may remain unchanged. Severe deformities with marked cosmetic or functional impairment may still require surgical correction. Further research involving larger sample sizes, objective functional outcome measures, and long-term follow-up is recommended to establish standardized rehabilitation guidelines for SD.

Strengths

This case report provides a detailed description of the clinical presentation, radiological findings, and physiotherapy management of a patient with mild Sprengel's deformity, a relatively rare congenital musculoskeletal condition. The study utilized a structured and progressive six-week rehabilitation program with clearly defined treatment phases. Objective outcome measures, including range of motion, Manual Muscle Testing (MMT), Numeric Pain Rating Scale (NPRS), and Disabilities of the Arm, Shoulder and Hand (DASH) scores, were used to assess treatment effectiveness. The findings contribute to the limited literature regarding conservative physiotherapy management of Sprengel's deformity and demonstrate the potential role of rehabilitation in improving shoulder function, scapular control, and overall upper-extremity performance.

Limitations

As this is a single case report, the findings should be interpreted within the context of an individual patient and may not be directly generalizable to all cases of Sprengel's deformity. The study evaluated outcomes over a six-week rehabilitation period, and longer-term follow-up could provide additional information regarding the sustainability of functional improvements. Nevertheless, the case offers valuable clinical insights into the potential benefits of a structured physiotherapy rehabilitation program and contributes to the limited evidence available on conservative management of Sprengel's deformity.

## Conclusions

The present case suggests that a structured physiotherapy rehabilitation program may contribute to improvements in functional limitations associated with mild Sprengel's deformity. Following a six-week rehabilitation protocol, improvements were observed in shoulder mobility, periscapular muscle strength, scapular stability, posture, and overhead functional performance. These findings indicate that conservative physiotherapy management may support enhanced upper-limb function and quality of life in individuals with mild Sprengel's deformity. However, given the single-case design, these observations should be interpreted with caution. Further studies with larger sample sizes and long-term follow-up are required to determine the generalizability of these findings and to establish standardized rehabilitation guidelines.
